# Assessment of Spanish Panel Reactive Antibody Calculator and Potential Usefulness

**DOI:** 10.3389/fimmu.2017.00540

**Published:** 2017-05-11

**Authors:** Esther Asensio, Marcos López-Hoyos, Íñigo Romón, Jesús Ontañón, David San Segundo

**Affiliations:** ^1^Immunology Service, Marqués de Valdecilla Universitary Hospital-IDIVAL, Santander, Cantabria, Spain; ^2^Tissue Typing Laboratory, Marqués de Valdecilla Universitary Hospital-IDIVAL, Santander, Cantabria, Spain; ^3^Immunology, Universitary Hospital of Albacete, Castilla la Mancha, Spain

**Keywords:** anti-human leukocyte antigen antibodies, calculated panel reactive of antibody, kidney transplantation, highly sensitized patients, organ allocation, PATHI

## Abstract

**Background and objectives:**

The calculated panel reactive of antibodies (cPRAs) necessary for kidney donor-pair exchange and highly sensitized programs are estimated using different panel reactive antibody (PRA) calculators based on big enough samples in Eurotransplant (EUTR), United Network for Organ Sharing (UNOS), and Canadian Transplant Registry (CTR) websites. However, those calculators can vary depending on the ethnic they are applied. Here, we develop a PRA calculator used in the Spanish Program of Transplant Access for Highly Sensitized patients (PATHI) and validate it with EUTR, UNOS, and CTR calculators.

**Methods:**

The anti-human leukocyte antigen (HLA) antibody profile of 42 sensitized patients on waiting list was defined, and cPRA was calculated with different PRA calculators.

**Results:**

Despite different allelic frequencies derived from population differences in donor panel from each calculator, no differences in cPRA between the four calculators were observed. The PATHI calculator includes anti-DQA1 antibody profiles in cPRA calculation; however, no improvement in total cPRA calculation of highly sensitized patients was demonstrated.

**Interpretation and conclusion:**

The PATHI calculator provides cPRA results comparable with those from EUTR, UNOS, and CTR calculators and serves as a tool to develop valid calculators in geographical and ethnic areas different from Europe, USA, and Canada.

## Introduction

Kidney transplantation of highly sensitized patients remains a challenge. The presence of numerous preformed anti-human leukocyte antigen (HLA) antibodies makes it difficult to find a compatible donor. Therefore, these patients spend prolonged periods of time on the waiting list with associated increase in morbidity and mortality (1).

The way to measure the sensitization level has traditionally been the panel reactive antibodies (PRAs). The PRA is determined by testing the patient’s serum against a panel of HLA-typed donor cells and estimating the likelihood of finding a crossmatch compatible donor. However, PRA is labor intensive and depends on the composition of the panel limited by the number of donors available and the techniques used for anti-HLA antibodies detection.

The introduction of solid-phase assays using single HLA antigens manufactured by recombinant DNA technologies (Luminex) has increased the sensitivity and specificity to detect and identify HLA-specific antibodies compared to previous methods like complement-dependent cytotoxicity (CDC) (2, 3). The improvement of sensitivity not only allows a better definition of the anti-HLA antibody profile or forbidden HLA antigens but also identifies permissive antigens for transplantation.

The virtual crossmatch (vXM) could anticipate the results of complement-dependent cytotoxic crossmatch comparing HLA-typed donors with acceptable HLA antigens defined by the patient anti-HLA antibody profile (4).

The calculated panel reactive of antibody (cPRA) is based upon unacceptable HLA antigens (UHA), identified by the presence of HLA-specific antibodies in the sera of transplant candidates. It is calculated from the HLA antigen frequencies in a given population and represents the percentage of actual kidney donors that express at least one of the unacceptable antigens.

The different organ allocation systems such as Eurotransplant (EUTR), United Network for Organ Sharing (UNOS), and Canadian Transplant Registry (CTR) try to increase the chances of highly sensitized patients to be transplanted by giving them additional points or including them in special programs and perform vXM in transplant alerts. To avoid skewing in the allocation, due to different allelic frequencies among geographical areas (5), every transplant program ideally should use its own donor database to calculate the cPRA.

In 2014 the Spanish Ministry of Health, through the National Transplant Organization implemented the Spanish Program of Transplant Access for Highly Sensitized patients (PATHI), a specific program for highly sensitized renal patients. Here, we present the cPRA results obtained with the PRA calculator setup for the program and compare them with established PRA calculators from international sources to ascertain their results for a given patient population.

## Materials and Methods

### PATHI Calculator

The PATHI calculator has been designed with 250 genotypes (HLA loci A, B, C, DRB1, DRB3, DRB4, DRB5, and DQB1) from donors typed in Tissue Typing Laboratory at University Hospital of Albacete, Spain, whereas the DQA1 was deducted by linkage disequilibrium (6–8). Unlike other systems using the formula developed by Zachary and Braun (9) based on population frequency of antigens and haplotypes, PATHI uses the classical system for PRA calculation dividing the number of positive reactions by the total number of subjects tested.

### Samples and Anti-HLA Testing

Serum samples from 42 sensitized patients on renal waiting list during 2015 from our institution (Hospital Universitario Marqués de Valdecilla) were studied for anti-HLA class I and class II antibody testing with the LABScreen^®^ Mixed and Single Antigen assays (One Lambda, Canoga Park, CA, USA) according to the manufacturer’s protocol. We considered as positive specificities when raw mean fluorescence intensity (MFI) above 1,500 and/or baseline MFI above 1,000.

### cPRA Comparison

Once the profile of UHA was defined for each patient, the cPRA was assessed using PATHI calculator for class I and class II antigens independently and for both classes combined. The results obtained were compared with those defined by cPRA from the EUTR and CTR webpage tool (10, 11) and the UNOS PRA calculator (12). The EUTR and UNOS calculators consider HLA-A, HLA-B, HLA-C, HLA-DR, and HLA-DQ frequencies whereas CTR and PATHI also include DQA1. The allele frequencies in PATHI calculator are shown in Table 1.

**Table 1:** **Allelic frequencies used for the panel reactive antibody PATHI^a^ calculator**.

A locus		B locus		DR locus	
A1	0.09226	B7	0.07696	DR1	0.08131
A2	0.27889	B8	0.05979	DR103	0.02020
A3	0.11456	B13	0.01005	DR2	0.12935
A11	0.07696	B18	0.08786	DR3	0.16021
A23 (9)	0.02635	B27	0.02635	DR4	0.12822
A24 (9)	0.10557	B35	0.11231	DR5	0.12485
A25 (10)	0.02225	B37	0.00000	DR6	0.14692
A26 (10)	0.04501	B38 (16)	0.03460	DR7	0.16813
A29 (19)	0.08131	B39 (16)	0.01005	DR8	0.01410
A30 (19)	0.06192	B41	0.01410	DR9	0.00602
A31 (19)	0.01207	B42	0.00200	DR10	0.01005
A32 (19)	0.01816	B44 (12)	0.17054	DR11 (5)	0.11682
A33 (19)	0.02225	B45 (12)	0.02430	DR12 (5)	0.00803
A34 (10)	0.00000	B46	0.00000	DR13 (6)	0.13282
A36	0.00000	B47	0.00401	DR14 (6)	0.01410
A43	0.00000	B48	0.00000	DR15 (2)	0.09889
A66 (10)	0.00401	B49 (21)	0.04501	DR16 (2)	0.03046
A68 (28)	0.02840	B50 (21)	0.02430	DR17 (3)	0.15620
A69 (28)	0.00602	B51 (5)	0.08131	DR18 (3)	0.00401
A74 (19)	0.00000	B52 (5)	0.01410		
A80	0.00200	B53	0.01207		
		B55 (22)	0.00401		
		B56 (22)	0.00200		
		B57 (17)	0.02635		
		B58 (17)	0.00803		
		B59	0.00000		
		B60 (40)	0.01005		
		B61 (40)	0.01005		
		B62 (15)	0.04711		
		B63 (15)	0.00401		
		B64 (14)	0.01005		
		B65 (14)	0.04292		
		B67	0.00000		
		B71 (70)	0.00000		
		B72 (70)	0.00200		
		B73	0.00000		
		B75 (15)	0.00000		
		B76 (15)	0.00000		
		B77 (15)	0.00000		
		B78	0.00200		
		B81	0.00000		
		B82	0.00000		

*^a^Data obtained from 250 Spanish donors (details in Section “Material and Methods”)*.

### Statistical Analysis

The Kruskal–Wallis and Spearman’s rho test were used to compare results from each calculator and to assess the correlation between the calculators. All the statistical analyses were performed using GraphPad Prism 5 software (San Diego, CA, USA).

## Results

**Figure 1:**
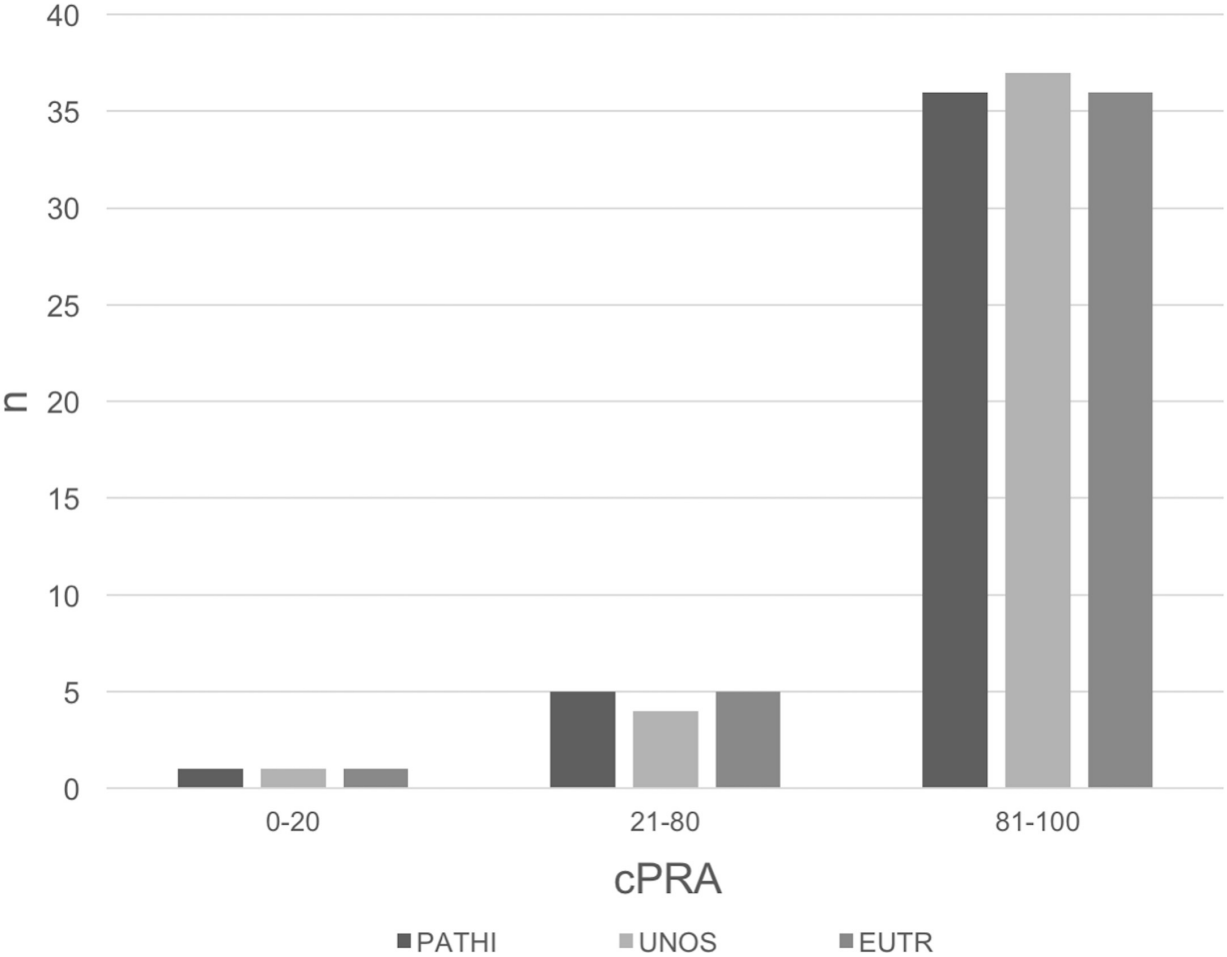
**Frequency distribution of calculated panel reactive of antibody (cPRA) with different panel reactive antibody calculators**. Different ranges of cPRA were established: 0–20% (low sensitized), 21–80% (sensitized), and 81–100% (highly sensitized).

The distribution of cPRA calculated with PATHI in the 42 sensitized patients in our waiting list is summarized in Figure 1 where 33 of 42 presented >90% total cPRA. The total cPRA was calculated with different PRA calculators (10–12), but no differences were observed between calculators (Table 2). The correlation of cPRA calculations between PATHI and the other calculators was significant (Figure S1 in Supplementary Material). These calculators allow the estimation of independent class I and class II cPRA, but no differences were found in individual cPRA for either class I or class II antibodies (Table 2).

**Table 2:** **Comparison of class I, class II, and total calculated panel reactive of antibody (cPRA) with different calculators**.

cPRA, median (IQR)	PATHI	United Network for Organ Sharing	Eurotransplant	CTR	*p* Value
Class I	91 (38.2–99)	89 (37.2–99)	89.8 (38.3–98.6)	89.5 (38–99)	0.99
Class II	86.5 (55.7–98)	92.5 (60.5–98.2)	92.4 (61.4–98.5)	89 (60.5–98)	0.90
Total	99 (94–100)	99 (95–100)	99 (94.6–99.9)	99 (94.7–100)	0.96

*IQR, interquartile range*.

*Kruskal–Wallis test was applied*.

Since PATHI calculator is used to evaluate sensitized patients in order to get access to the highly sensitized program, we compared the total cPRA in those patients with cPRA >90% (Figure S2 in Supplementary Material). A slightly better correlation of total cPRA including only these highly sensitized patients between PATHI and EUTR calculator was observed (*r* = 0.90 vs 0.87 vs 0.88, UNOS and CTR, respectively).

The main difference between PATHI and EUTR and UNOS calculators is that it includes HLA-DQA1 antigens. To assess the potential impact of HLA-DQA1 antigens in cPRA calculation, we compared the cPRA obtained with only DQA1 profile reaction and their DQB1 (Table 3) and DRB1 associations (Table S1 in Supplementary Material). The cPRA is much lower when DQA1 immunization is considered. For example, when a patient reacts against DQA1*02 and the calculator lacks DQA1 antigens, the assignment of DQB1 associations should be included as UHA (i.e., DQB1*02, DQB1*03, and DQB1*04). Consequently, the cPRA increases from 31% (PATHI-DQA1) to 83, 80.48, and 83% (in PATHI without DQA1, EUTR, and UNOS calculators, respectively, Table 3).

**Table 3:** **Comparison of calculated panel reactive of antibody (cPRA) assessing the effect of DQA1 anti-human leukocyte antigen profile and their associated DQB1 alleles**.

DQA1 unique reactive profile	cPRA PATHI (DQA1) (%)	Associated DQB1	cPRA PATHI (%)	cPRA Eurotransplant (%)	cPRA United Network for Organ Sharing (%)
*01	58	*05, *06	58	68.37	64
*02	31	*02, *03, *04	83	80.48	83
*03	26	*02, *03, *04	83	80.48	83
*04	3	*02, *04	55	39.75	45
*05	50	*02, *03:01	70	78.15	67
*06	0	*03:01	30	56.16	39

To further investigate the impact on different cPRA calculation including the DQA1-typed donors, we studied highly sensitized patients with anti-HLA antibody profile of DQA1 reaction. Three of 33 highly sensitized patients had different degrees of DQA1 reactivity (summarized in Table 4).

**Table 4:** **Cases of anti-human leukocyte antigen (HLA) antibody profile against DQA1 and the effect on DQ, class II, and global calculated panel reactive of antibody (cPRA)**.

Case number	Specificities					cPRA								
	Anti-HLA class I antibodies		Anti-HLA class II antibodies			DQ cPRA			Class II cPRA			Total cPRA		
	A antigens	B antigens	DR antigens	DQB1	DQA1	PATHI	Eurotransplant (EUTR)	United Network for Organ Sharing (UNOS)	PATHI	EUTR	UNOS	PATHI	EUTR	UNOS
Case 1				*03:01, *04	*02, *03	71			92			100		
	1, 2, 23, 24, 25, 26, 29, 31, 32, 33, 34, 36, 43, 66, 68, 69, 74, 80	13, 35, 38, 44, 45, 46, 49, 50, 51, 52, 53, 56, 57, 58, 59, 62, 63, 73, 75, 76, 77, 82	1, 4, 7, 9, 10, 51, 53, 103	*02, *03, *04		83	80.48	83	98	97.83	97	100	99.96	100

Case 2				*02, *03:01, *05	*02	86			95			100		
	1, 3, 11, 24, 25, 26, 29, 30, 31, 32, 33, 34, 36, 43, 66, 68, 69, 74, 80	7, 8, 13, 18, 27, 37, 38, 39, 41, 42, 44, 45, 46, 47, 48, 49, 51, 52, 53, 54, 55, 56, 57, 58, 59, 60, 61, 62, 63, 64, 65, 67, 71, 72, 73, 75, 76, 77, 78, 81, 82	1, 7, 9, 10, 12, 52, 103	*02, *03, *04, *05		94	94.12	96	98	98.41	99	100	100	100

Case 3				*03, *04, *05	*03	72			93			100		
	1, 2, 3, 11, 24, 25, 26, 29, 30, 31, 33, 34, 36, 43, 66, 68, 69, 74, 80	7, 13, 18, 27, 35, 37, 41, 42, 49, 51, 52, 53, 54, 55, 56, 57, 58, 59, 67, 73, 78, 81, 82	1, 4, 7, 8, 9, 10, 13, 14, 53, 103	*02, *03, *04, *05		94	94.12	96	98	98.41	99	100	100	100

The class II cPRA calculated with serologic DQ antigens was higher than with PATHI-DQA1 calculator: Case #1: 98, 97.83, and 97 vs 92%; Case #2: 98, 98.41, and 99 vs 95%, and Case #3: 98, 98.41, and 99 vs 93% (PATHI, EUTR, and UNOS vs PATHI-DQA1 calculators). Nevertheless, the reduction in the class II cPRA was abolished when total cPRA was estimated in highly sensitized patients: Case #1: 100, 99.96, and 100 vs 100%; Case #2: 100, 100, and 100 vs 100%, and Case #3: 100, 100, and 100 vs 100% (PATHI, EUTR, and UNOS vs PATHI-DQA1). Moreover, the use of both DQA1-DRB1 and DQA1-DQB1 association showed comparable results of cPRA between the calculators in highly sensitized patients (Table S2 in Supplementary Material).

## Discussion

An accurate calculation of the cPRA is crucial for highly sensitized patients to be included in special allocation programs that increase their chances to be offered an organ. Different calculators have been developed with this purpose. The EUTR group established the Acceptable Mismatch program for patients with a cPRA >85% and at least 2 years on dialysis. The new Organ Procurement and Transplantation Network Kidney Allocation Scheme gives the highest priority for local, regional, and national sharing to candidates with cPRA values of 98, 99, and 100% respectively. In Spain, PATHI includes potential recipients with a cPRA >98% and at least 1 year on dialysis with additional points according to the time in dialysis, age, and distance between donation and transplant center.

The EUTR PRA calculator uses HLA typing data from 6,870 organ donors. The UNOS cPRA is determined using an established logarithm and based on ethnic frequencies and HLA frequencies derived from the HLA phenotypes of more than 14,000 deceased donors whereas CTR has more than 1,700 donors in 2011 (13).

PATHI calculator takes into account the HLA phenotypes of 250 donors. This seems to be a major limitation of our calculator. Nonetheless, its intended use is to serve as a tool to implement a cPRA calculator in those geographic areas with special HLA alleles in which the use of EUTR or UNOS calculator cannot be applied. We are aware that the frequency of some of the HLA antigens could be over or underestimated due to the small number of donors included, but the percentages of positive reactions seems not be skewed by sample size of donor panel, as recently discussed (14). However, with 250 typed donors, it is possible to obtain representative allelic frequencies of the population although the number would be insufficient in calculating haplotype frequencies that could not be used for the calculation of PRA according to the frequencies. Nevertheless, the classic PRA calculation method is based on crossmatch with panels of 30–50 cells; therefore, the method based on vXM significantly increases the sample size of the standard reference sample.

The PATHI calculator is very flexible and can be easily adapted to have a more accurate representation of a donor population by introducing the HLA typing of new donors.

Another potential advantage of PATHI calculator is the inclusion of HLA-DQA1, which is not considered in EUTR and UNOS calculators but included in the Canadian calculator from CTR (13). Unlike PATHI, DQA1 data of CTR are from typed donors, comparable cPRAs were found (Table 2; Figures S1 and S2 in Supplementary Material). These results could validate the use of linkage-based data to “real” HLA typing data in the formulation of new calculators. Although HLA-DQA1 typing is not usually performed in tissue typing laboratories, it would be nowadays advisable in those countries with active kidney donor-pair exchange and highly sensitized programs.

This fact is of the utmost importance, especially in the case of patients whose serum reacts clearly with HLA-DQA1 and not with HLA-DQB1 antigens. The cPRA can change considerably when considering only anti-HLA-DQA1 instead of anti-HLA-DQ antibodies (Table 4), although in highly sensitized patients similar cPRAs were obtained irrespectively of DQA1-DQB1 or DQA1-DRB1 association applied in the calculator (Table S2 in Supplementary Material). In cases of highly sensitized patients, the class I antigen specificities could overcome the improvement of PRA calculators that include DQA1-typed donors. Nevertheless, the importance of calculators based on DQA1-typed donors over total cPRA in highly sensitized patients reacting only with class II antigens should be considered, especially when PRA cutoff values are used as the main criterion to include highly sensitized patients in donor-pair exchange and highly sensitized programs. In fact, recently, a reduction in cPRA using a modified UNOS calculator including DQA1 antigens vs UNOS calculator was observed (15).

None of the calculators assessed takes into account the anti-HLA-DP antigens. Despite low grade of polymorphism of HLA-DP, the inclusion of DPA and DPB antigens in CTR has resulted in an increase in cPRA (14). It remains to be tested with our and other calculators.

The differences observed in PRA with the calculators may be due to the differences in the frequency of each antigen in the reference population rather than the method of calculation.

The PRA calculator used for allocation should be accurate enough to avoid positive CDC crossmatch with previous vXM negative but also have to define UHA precisely in order to avoid delayed time in organ allocation. The PATHI calculator for highly sensitized patients obtained the same results as UNOS and EUTR PRA calculators, offering a consistent tool to allocate highly sensitized patients in our program and demonstrating its potential use in other geographical settings with small numbers.

## Ethics Statement

This study was carried out in accordance with the recommendations of Ethical Committee of Cantabria with written informed consent from all subjects. All subjects gave written informed consent in accordance with the Declaration of Helsinki.

## Author Contributions

EA: contributed with drafting the work and the acquisition, analysis, and interpretation of the data. ML-H and JO: contributed with the interpretation of the data, revising and final approval of the version published. IR: contributed with the design of the work, analysis of the data, and drafting the work. DS: contributed with the design of the work, drafting the work, interpretation of the data, and final approval of the version published.

## Conflict of Interest Statement

The authors declare that the research was conducted in the absence of any commercial or financial relationships that could be construed as a potential conflict of interest.

## Supplementary Material

Figure S1 **Comparison of calculated panel reactive of antibody (cPRA) with different calculators**. The cPRA measured with PATHI calculator was compared with United Network for Organ Sharing (UNOS) **(A)**, Eurotransplant (EUTR) **(B)**, and CTR **(C)** calculators.

Figure S2 **Correlation of calculated panel reactive of antibody (cPRA) measured with different calculators in highly sensitized patients**. The cPRA comparison was performed in patients with cPRA above 90%. The cPRA measured with PATHI calculator was compared with United Network for Organ Sharing (UNOS) **(A)**, Eurotransplant (EUTR) **(B)**, and CTR **(C)** calculators.
