# Supplementary material for: Assessment of Spanish Panel Reactive Antibody Calculator and Potential Usefulness
Source: Front Immunol. 2017 May 11;8:540. doi: 10.3389/fimmu.2017.00540 (PMC5425465; doi:10.3389/fimmu.2017.00540)
Supplement: Supplementary file 1 [file Table_1.DOCX]

**Supplementary Table 1. Comparison of cPRA assessing the effect of DQA1 anti-HLA profile and their associated serologic DRB1 antigens.**

| **DQA1 unique reactive profile** | **cPRA PATHI (DQA1)** | **Associated serologic DRB1** | **cPRA PATHI** | **cPRA EUTR** | **cPRA UNOS** |
| --- | --- | --- | --- | --- | --- |
| *01 | 58% | *01,*103,*04,*08,*10,*11,*12,*13,*14,*15,*17 | 95% | 97.35% | 97% |
| *02 | 31% | *07,*13 | 52% | 43.96% | 41% |
| *03 | 26% | *04,*08,*09,*11,*12,*13,*14,*15 | 78% | 85.14% | 87% |
| *04 | 3% | *08,*18 | 4% | 6.21% | 11% |
| *05 | 50% | *103,*11,*12,*13,*14,*16,*17,*18 | 75% | 70.33% | 65% |
| *06 | 0% | *08 | 3% | 6.19% | 9% |
