# Supplementary material for: Assessment of Spanish Panel Reactive Antibody Calculator and Potential Usefulness
Source: Front Immunol. 2017 May 11;8:540. doi: 10.3389/fimmu.2017.00540 (PMC5425465; doi:10.3389/fimmu.2017.00540)
Supplement: Supplementary file 2 [file Table_2.DOCX]

**­­Supplementary Table 2. Comparison of DQA1-DQB1 and DQA1-DRB1 associations in cPRA of highly-sensitized patients**

|  | **Case number** | **Anti-HLA Class-I antibodies** | | **Anti-HLA Class-II antibodies** | | | | | | **Class-II cPRA** | | | **Total cPRA** | | |
| --- | --- | --- | --- | --- | --- | --- | --- | --- | --- | --- | --- | --- | --- | --- | --- |
|  |  | **A antigens** | **B antigens** | **DQA1** | **DQB1** | **DRB1** | **DRB3** | **DRB4** | **DRB5** | **PATHI** | **EUTR** | **UNOS** | **PATHI** | **EUTR** | **UNOS** |
| **Association** |  | **Specificities** | | | | | | | | **cPRA** | | | | | |
|  | Case 1 |  |  |  |  |  |  |  |  |  |  |  |  |  |  |
|  |  |  |  | *02, *03 | *03:01 (DQ7), *04 | *01, *04, *07, *09, *10, *103 | - | *01 (DR53) | *01, *02 (DR51) | 92 | - | - | - | - | - |
| DQA1  DQB1 |  |  |  |  | *02,*03,*04 | *01, *04, *07, *09, *10, *103 | - | *01 (DR53) | *01, *02 (DR51) | 98 | 97.83 | 97 | - | - | - |
| DQA1  DRB1 |  |  |  |  | *03:01 (DQ7), *04 | *01, *04, *07, *08, *09, *10, *11, *12, *13, *14, *15 *103 | - | *01 (DR53) | *01, *02 (DR51) | 98 | 98.95 | 99.31 | - | - | - |
|  |  | *01, *02, *23, *24, *25, *26, *29, *31, *32, *33, *34, *36, *43, *66, *68, *69, *74, *80 | *13, *35, *38, *44, *45, *46, *49, *50, *51, *52, *53, *56, *57, *58, *59, *62, *63, *73, *75, *76, *77, *82 | *02, *03 | *03:01 (DQ7), *04 | *01, *04, *07, *09, *10, *103 | - | *01 (DR53) | *01, *02 (DR51) | - | - | - | 100 | - | - |
| DQA1  DQB1 |  | *01, *02, *23, *24, *25, *26, *29, *31, *32, *33, *34, *36, *43, *66, *68, *69, *74, *80 | *13, *35, *38, *44, *45, *46, *49, *50, *51, *52, *53, *56, *57, *58, *59, *62, *63, *73, *75, *76, *77, *82 |  | *02,*03,*04 | *01, *04, *07, *09, *10, *103 | - | *01 (DR53) | *01, *02 (DR51) | - | - | - | 100 | 99.96 | 100 |
| DQA1  DRB1 |  | *01, *02, *23, *24, *25, *26, *29, *31, *32, *33, *34, *36, *43, *66, *68, *69, *74, *80 | *13, *35, *38, *44, *45, *46, *49, *50, *51, *52, *53, *56, *57, *58, *59, *62, *63, *73, *75, *76, *77, *82 |  | *03:01 (DQ7), *04 | *01, *04, *07, *08, *09, *10, *11, *12, *13, *14, *15 *103 | - | *01 (DR53) | *01, *02 (DR51) | - | - | - | 100 | 99.98 | 99.99 |
|  | Case 2 |  |  |  |  |  |  |  |  |  |  |  |  |  |  |
|  |  |  |  | *02 | *02,*03:01 (DQ7), *05 | *01, *07, *09, *10, *12, *103 | *01,*02,*03  (DR52) | - | - | 95 | - | - | - | - | - |
| DQA1  DQB1 |  |  |  |  | *02,*03,*04,*05 | *01, *07, *09, *10, *12, *103 | *01,*02,*03  (DR52) | - | - | 98 | 98.41 | 99 | - | - | - |
| DQA1  DRB1 |  |  |  |  | *02,*03:01 (DQ7), *05 | *01, *07, *09, *10, *12, *13 *103 | *01,*02,*03  (DR52) | - | - | 95 | 97.63 | 96.42 | - | - | - |
|  |  | *01, *03, *11, *24, *25, *26, *29, *30, *31, *32, *33, *34, *36, *43, *66, *68, *69, *74, *80 | *07, *08, *13, *18, *27, *37, *38, *39, *41, *42, *44, *45, *46, *47, *48, *49, *51, *52, *53, *54, *55, *56, *57, *58, *59, *60, *61, *62, *63, *64, *65, *67, *71, *72, *73, *75, *76, *77, *78, *81, *82 | *02 | *02,*03:01 (DQ7), *05 | *01, *07, *09, *10, *12, *103 | *01,*02,*03  (DR52) | - | - | - | - | - | 100 | - | - |
| DQA1  DQB1 |  | *01, *03, *11, *24, *25, *26, *29, *30, *31, *32, *33, *34, *36, *43, *66, *68, *69, *74, *80 | *07, *08, *13, *18, *27, *37, *38, *39, *41, *42, *44, *45, *46, *47, *48, *49, *51, *52, *53, *54, *55, *56, *57, *58, *59, *60, *61, *62, *63, *64, *65, *67, *71, *72, *73, *75, *76, *77, *78, *81, *82 |  | *02,*03,*04,*05 | *01, *07, *09, *10, *12, *103 | *01,*02,*03  (DR52) |  |  | - | - | - | 100 | 100 | 100 |
| DQA1  DRB1 |  | *01, *03, *11, *24, *25, *26, *29, *30, *31, *32, *33, *34, *36, *43, *66, *68, *69, *74, *80 | *07, *08, *13, *18, *27, *37, *38, *39, *41, *42, *44, *45, *46, *47, *48, *49, *51, *52, *53, *54, *55, *56, *57, *58, *59, *60, *61, *62, *63, *64, *65, *67, *71, *72, *73, *75, *76, *77, *78, *81, *82 |  | *02,*03:01 (DQ7), *05 | *01, *07, *09, *10, *12, *13 *103 | *01,*02,*03  (DR52) | - | - | - | - | - | 100 | 100 | 99.97 |
|  | Case 3 |  |  |  |  |  |  |  |  |  |  |  |  |  |  |
|  |  |  |  | *03 | *03,*04,*05 | *01, *04, *07, *08, *09, *10, *13, *14, *103 | - | *01 (DR53) | - | 93 | - | - | - | - | - |
| DQA1  DQB1 |  |  |  |  | *02,*03,*04,*05 | *01, *04, *07, *08, *09, *10, *13, *14, *103 | - | *01 (DR53) | - | 98 | 98.41 | 99 | - | - | - |
| DQA1  DRB1 |  |  |  |  | *03,*04,*05 | *01, *04, *07, *08, *09, *10,*11,*12, *13, *14, *15, *103 | - | *01 (DR53) | - | 98 | 98.84 | 99.62 | - | - | - |
|  |  | *01, *02, *03, *11, *24, *25, *26, *29, *30, *31, *33, *34, *36, *43, *66, *68, *69, *74, *80 | *07, *13, *18, *27, *35, *37, *41, *42, *49, *51, *52, *53, *54, *55, *56, *57, *58, *59, *67, *73, *78, *81, *82 | *03 | *03,*04,*05 | *01, *04, *07, *08, *09, *10, *13, *14, *103 | - | *01 (DR53) | - | - | - | - | 100 | - | - |
| DQA1  DQB1 |  | *01, *02, *03, *11, *24, *25, *26, *29, *30, *31, *33, *34, *36, *43, *66, *68, *69, *74, *80 | *07, *13, *18, *27, *35, *37, *41, *42, *49, *51, *52, *53, *54, *55, *56, *57, *58, *59, *67, *73, *78, *81, *82 |  | *02,*03,*04,*05 | *01, *04, *07, *08, *09, *10, *13, *14, *103 | - | *01 (DR53) | - | - | - | - | 100 | 100 | 100 |
| DQA1  DRB1 |  | *01, *02, *03, *11, *24, *25, *26, *29, *30, *31, *33, *34, *36, *43, *66, *68, *69, *74, *80 | *07, *13, *18, *27, *35, *37, *41, *42, *49, *51, *52, *53, *54, *55, *56, *57, *58, *59, *67, *73, *78, *81, *82 |  | *03,*04,*05 | *01, *04, *07, *08, *09, *10,*11,*12, *13, *14, *15, *103 | - | *01 (DR53) | - | - | - | - | 100 | 100 | 100 |
