# Supplementary figures and images for: Assessment of Spanish Panel Reactive Antibody Calculator and Potential Usefulness
Source: Front Immunol. 2017 May 11;8:540. doi: 10.3389/fimmu.2017.00540 (PMC5425465; doi:10.3389/fimmu.2017.00540)

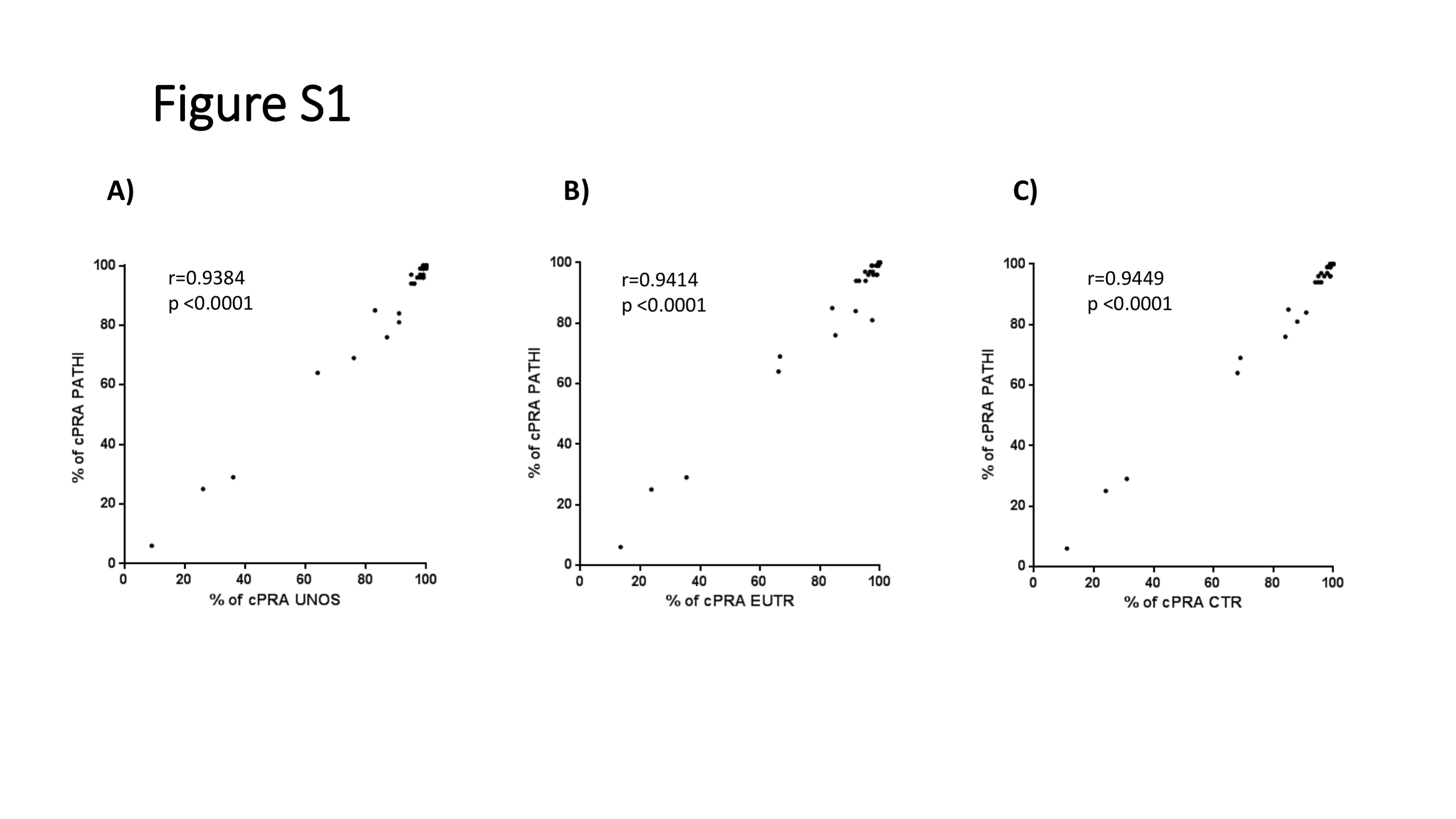

Supplement: Figure S1 — Comparison of calculated panel reactive of antibody (cPRA) with different calculators. The cPRA measured with PATHI calculator was compared with United Network for Organ Sharing (UNOS) (A), Eurotransplant (EUTR) (B), and CTR (C) calculators. [file Image_1.PNG]

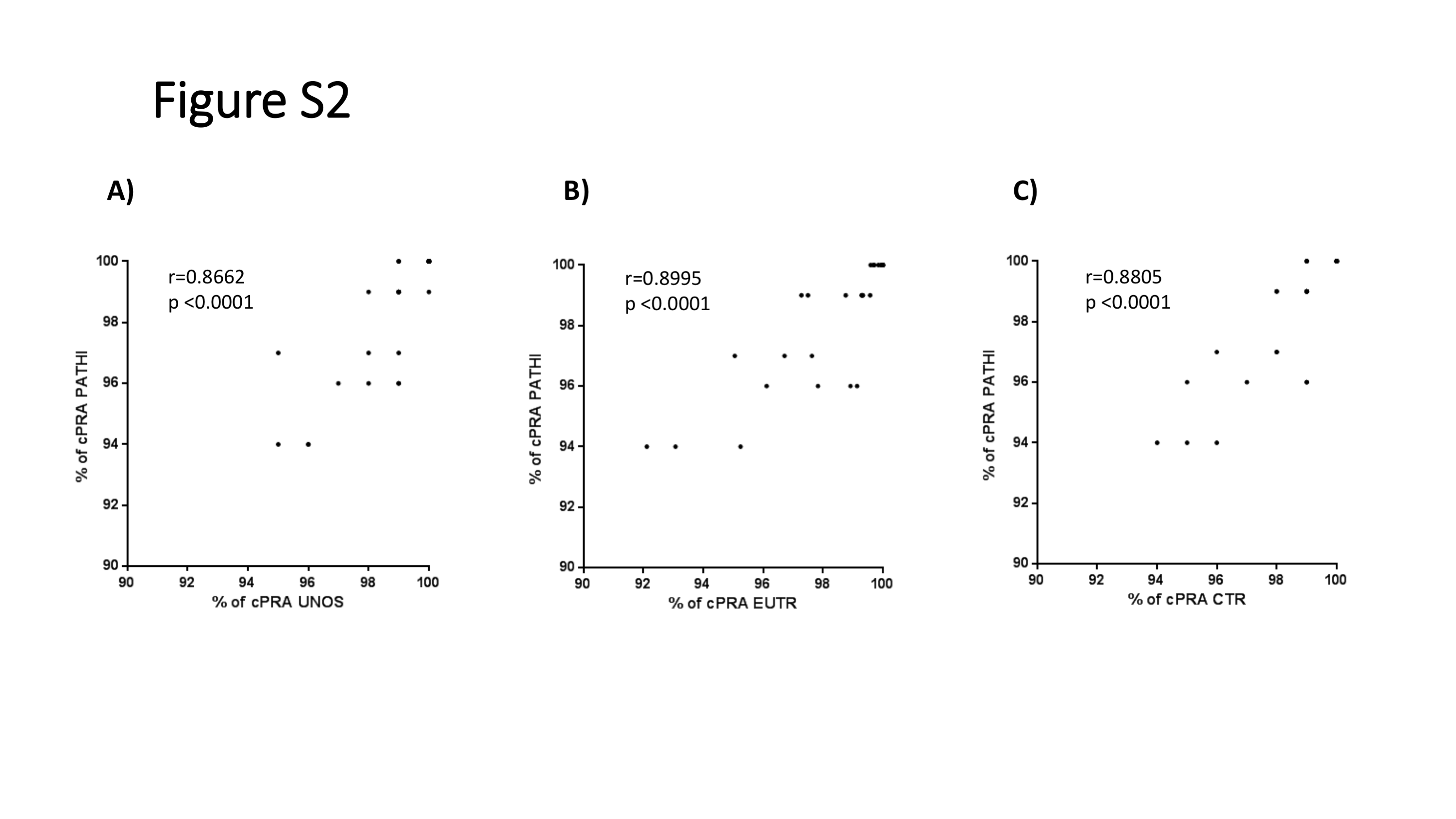

Supplement: Figure S2 — Correlation of calculated panel reactive of antibody (cPRA) measured with different calculators in highly sensitized patients. The cPRA comparison was performed in patients with cPRA above 90%. The cPRA measured with PATHI calculator was compared with United Network for Organ Sharing (UNOS) (A), Eurotransplant (EUTR) (B), and CTR (C) calculators. [file Image_2.PNG]
